# Starting from scratch: prevalence, methods, and functions of non-suicidal self-injury among refugee minors in Belgium

**DOI:** 10.1186/s13034-018-0260-1

**Published:** 2018-12-18

**Authors:** Sarah Verroken, Chris Schotte, Ilse Derluyn, Imke Baetens

**Affiliations:** 1 Faculty of Psychology and Educational Sciences, Research Group Lifespan and Clinical Psychology, VUB PE KLEP, Vrije Universiteit Brussel, Pleinlaan 2, 1050 Brussels, Belgium; 2 Department Clinical Psychology, UZ Brussel, Jette, Belgium; 3 Faculty of Psychology and Educational Sciences, Department of Social Work and Social Pedagogy, Centre for the Social Study of Migration and Refugees, Universiteit Gent, Henri Dunantlaan 2, 9000 Ghent, Belgium

**Keywords:** Non-suicidal self-injury, NSSI, Refugee minors, Prevalence, Methods, Functions

## Abstract

**Background:**

As many refugee minors have gone/go through stressful life experiences and uncertainty, one might expect mental health issues, including self-injury. However, literature on non-suicidal self-injury (NSSI) in refugee minors is scarce. This study explores the prevalence, methods, and functions of NSSI in refugee minors in Belgium, and compares research results to the existing literature on NSSI in Western adolescents.

**Methods:**

Data were obtained from 121 refugee minors (mean age = 16.12, SD = 1.23; range 14–18 years) through schools located in the Flemish and Brussels-Capital regions of Belgium. The sample consists of 39.7% girls and 60.3% boys. Self-report questionnaires were used to explore socio-economic data, NSSI behaviour (e.g. The Brief Non-Suicidal Self-injury Assessment Tool; BNNSI-AT) and emotional and behavioural difficulties (The Strengths and Difficulties Questionnaire; SDQ). Non-parametric Chi square tests were used for statistical comparisons of the obtained data as well as independent-sample t-tests and Fisher’s exact tests.

**Results:**

Results show a lifetime NSSI prevalence rate of 17.4%. Being accompanied or not, having both parents around, or living in an asylum centre did not influence NSSI prevalence. An average of 2.65 methods of NSSI was applied (SD = 2.50; range 1–9). The mean number of functions per person was six (*SD *= 4.97, range 0–16), with automatic functions reported the most. The data do point towards a greater psychological strain, with 68.4% reporting more than five acts of NSSI. Results of the SDQ’s Total Difficulties Scale and, more specifically, of the Emotional Problems, Conduct Problems, Peer Problems and Impact Scales indicate a substantial risk of clinically significant problems within the NSSI group. The Peer Problems and Impact Scales also point towards a high risk for suicidality amongst self-injuring refugees.

**Conclusions:**

Prevalence rates, methods and functions are comparable to Western samples. However, the higher incidence of the NSSI and the results on the SDQ also emphasise the vulnerability of refugee minors.

## Background

The Geneva convention defines a ‘refugee’ as “someone who is unable or unwilling to return to their country of origin owing to a well-founded fear of being persecuted for reasons of race, religion, nationality, membership of a particular social group, or political opinion” [1]. Unaccompanied minors are less than 18 years of age, not accompanied by any person exercising parental authority or custody under the national law of the minor, and originating from countries other than those in the European Economic Area [2]. The above definition of ‘refugee’ implies that most of them have experienced insecurity and stressful or even traumatic life events in their country of origin. Adverse life events [e.g. 3, 4], psychological distress [e.g. 5, 6], identity confusion [e.g. 7], and ethnic status [e.g. 8] are all risk factors for non-suicidal self-injury (NSSI) that can be expected in the majority of refugee minors. However, research on NSSI in refugee adolescents is scarce. Consequently, the primary aim of this study is to situate NSSI within a refugee adolescent population.

Refugee minors who have been exposed to war and political violence report traumatic loss, bereavement, separation, forced displacement, community and domestic violence, physical abuse, emotional abuse, impairment in the caregiver’s caregiving performance, etcetera [9]. During their transit, numerous stressful and dangerous situations may have occurred. Once they have arrived at their destination, a long asylum procedure, a difficult integration and an uncertain future await [e.g. 10–13]. Due to different origins, ethnicities, cultures, family and personal histories, refugees constitute a diverse, heterogeneous group with increased levels of psychological distress as a common factor. More specifically, post-traumatic stress disorder, depression and anxiety disorders are frequently reported in refugee children [9, 11, 14]. Despite pre- and post-migration distress, young refugees, like other adolescents, begin to develop a personal identity. Rejection by peers of the same ethnicity is an obstacle to this development. The integration of racial and ethnic identities into new social and cultural contexts might complicate this already demanding process, causing acculturative distress [9, 15, 16].

In comparison to Belgian adolescents, peers with a migration background report significantly more traumatic events, symptoms of severe post-traumatic stress, as well as higher avoidance scores. They do, however, show less anxiety symptoms and comparable amounts of depressive and emotional symptoms. The amount of traumatic experiences influences the prevalence of emotional and behavioural problems [10]. Migrant adolescents report less externalising problems and lower hyperactivity scores than their Belgian counterparts [10, 17], as well as very low levels of high-risk behaviours (sexual risk taking, running away, etc.), crime involvement, and alcohol abuse, common in Western traumatized samples [9]. One possible explanation could be their striving for a better future [10, 17]. However, when parents or social workers are questioned about adolescent refugees’ emotional and behavioural problems, the refugee group scores significantly higher on both internalising and externalising problems than natives do [18]. These differing findings could suggest that the behaviour of refugee minors is either perceived as more problematic by others than by themselves or that refugee minors underestimate or underreport their own problems.

A literature review on self-harm in refugees and asylum seekers found that the hopelessness and loss of future aspirations, combined with a traumatic background, common in refugees, is a risk factor for self-harm [19].

A negative association was determined between engagement in NSSI at some point in life and levels of affirmation, belonging, and commitment to one’s ethnic group. Therefore, a sense of belonging could be seen as a protective factor against engagement in NSSI, but other factors such as socioeconomic status (SES) and gender, might influence aforementioned relationships [8, 20]. While ethnic/racial identity (being aware of and understanding social/historical/cultural aspects of one’s ethnic group) might be a protective factor, ethnic status is a significant predictor of non-suicidal self-injurious behaviour [8]. Religion, especially Baptism and Islam, serves as a protective factor [8, 21].

Unaccompanied refugee adolescents report more emotional problems, more symptoms of anxiety, more depression, and more post-traumatic stress reactions than those living with their parents in the country of asylum [10, 22, 23]. One study comparing the inpatient psychiatric care between accompanied and unaccompanied refugee minors found that the latter exhibited more self-harm and suicidal behaviour [23]. Jensen et al. [24] found that 11% of unaccompanied refugee minors displayed suicidal ideation. However, even though unaccompanied Afghan refugee minors are all likely to have lived through a range of traumatic experiences, only 34% reported clinical levels of PTSD in a study by Bronstein et al. [25] in comparison to the .4 to 10% measured in the general population. The mere fact that they succeeded in their flight could be an indication of their resilience and capacities [13, 18]. Bhui and colleagues [26] also hypothesised that people with certain mental disorders, such as psychosis, are less likely to succeed in their flight to a safer country. It might indeed be that only the strongest and most resilient of refugees make it to the Western world.

Wester and Trepal [20] found a sense of belonging to be negatively related to the number of methods reported. No significant differences are found between ethnic groups (Caucasian, African American, Hispanic, Asian American, and multiracial groups) concerning the number of methods used in NSSI [20].

In Western studies on adolescents, automatic reinforcement functions are reported more frequently than social reinforcement functions [27]. It is unclear whether this also applies on non-Western samples. A study on Hong Kong adolescents, for example, found the regulation of interpersonal issues to be the main function while NSSI did not serve to regulate negative emotions. Another study on university students in India found that the function of minor forms of NSSI is to regulate social environments by means of avoidance, while the function of moderate to severe NSSI serves to regulate emotions [8].

Research on non-suicidal self-injury has been predominantly conducted on White samples in Western countries [8]. (Western) adolescents engaging in NSSI show higher levels of psychological symptoms than not self-injurious youths [6]. Approximately one out of five young adults engaging in NSSI exhibit high clinical symptomatology [28]. Psychological distress measured at age 12 is considered a significant predictor of NSSI [5]. Many studies link adverse life events and trauma symptoms to self-injurious behaviour [e.g. 3, 4]. Literature on (non-clinical) Western adolescents reports a lifetime NSSI prevalence of 17.2–18% [29, 30], and a 12-month prevalence of 9.6% to 28.4% [30]. Research demonstrates equivalency across gender [30]. As one singular episode is sufficient for being included in the lifetime prevalence statistics, some studies differentiate between the more common occasional (e.g. one to four reported lifetime episodes) forms and repetitive forms of self-injury. The American Psychiatric Association proposes a minimum of five occasions in the last year as one of the DSM-5 criteria for ‘nonsuicidal self-injury’ [31]. Zetterqvistet al. found that 6.7% of adolescents in a community sample meet the DSM-5 criteria for an NSSI disorder diagnosis [32]. In two studies by Brunner, approximately one out of every four adolescents engaging in ‘deliberate self-injurious behaviour’ or D-SIB (without suicidal intent), did so on a repetitive basis (i.e. five or more instances), rather than occasionally. Repetitive self-injury is related to a greater psychological burden [33, 34].

Many adolescents (39.8–47.75%) restrict themselves to one singular method of NSSI (e.g. cutting, burning, etc.), although 11.26% to 22.8% apply four methods or more [34–36]. Females tend to prefer methods like scratching and cutting to punching objects with the intention of hurting themselves. The latter is more common in male subjects [37]. An average of 4.3 NSSI functions per adolescent was found by Zetterqvist et al. [4].

Automatic reinforcement functions (e.g. to feel something or to relieve tension) are reported more commonly than social reinforcement functions (e.g. to avoid activities or to get help) [4, 27, 38].

Although NSSI is non-suicidal in its primary intention, research does link NSSI to suicidal thoughts and behaviours [39–41]. There is a high co-morbidity in adolescence. NSSI is seen as a significant risk factor for suicidal ideation, with an almost threefold risk for suicidality after even one act of NSSI [40]. Suicidal ideation has been associated with automatic functions, as well as with the number of methods used and the urge of self-injury [41]. In their review, Hamza et al. [39] distil several studies on NSSI and suicide into three theories: (1) the ‘Gateway Theory’, which places NSSI and suicide as extremes on a continuum on which NSSI may build up towards committing suicide, (2) the ‘Third Variable Theory’, in which a third variable (e.g. psychiatric disorder or psychological distress) is responsible for both the engagement in NSSI and the suicidal behaviour, instead of NSSI increasing the suicidal risk, and (3) ‘Joiner’s Theory of Acquired Capability for Suicide’, in which NSSI can be seen as one of many means to practise suicide by learning to overcome the fear and pain associated with it. However, in Joiner’s Theory, other conditions have to be fulfilled as well for NSSI to result in suicide (i.e. social isolation and the feeling of being a burden to others). According to Whitlock and colleagues [40], focusing on enhancing the perceived meaning in life and positive relationships with others could reduce the risk of NSSI behaviour developing into suicidal thoughts or actions.

Research exploring non-suicidal self-injurious behaviour in adolescent refugees seems to be scarce, though some research points to cultural differences. The primary aim of this study is to explore the prevalence of NSSI behaviour within a refugee minor population, as well as the methods used and the functions ascribed to it. This study also aims to compare the results with existing literature on the Western adolescent population.

As many of the risk factors previously described (e.g. adverse life events, psychological distress, identity confusion, ethnic status, lower SES) can be applied to refugee minors, we hypothesize that the prevalence of NSSI for this population will exceed the prevalence known for their Western adolescent counterparts. We also hypothesize a higher prevalence of self-injurious behaviour within the group living without parents. We predict no gender differences in NSSI prevalence.

Studies on non-Western populations show no differences in the number of methods used for NSSI between samples of differing ethnicities. We therefore hypothesize similar numbers used by adolescent refugees. As research on methods and functions in non-Western populations is limited and indecisive, we will also compare methods and functions of NSSI common in refugee minors with the existing literature on Western adolescent samples.

## Methods

### Recruitment

When children between the ages of 12 and 18 move to Flanders (or the Dutch-speaking community in Brussels) from a non-Dutch speaking country (provided that Dutch is not their mother tongue) they are first sent to OKAN-schools. These schools, freely translated as ‘intensive language schools for non-Dutch speaking newcomers’, prepare children to be able to participate in regular education after 1 year. Education is compulsory in Belgium until the age of 18. As a result, all non-Dutch speaking migrant children, including refugee adolescents, will pass through these schools, regardless of their origin, parental situation, housing situation, etc. Doing research within these schools, instead of in asylum centres, maximises the heterogeneity of the participants. Six OKAN-schools located in the Flemish and Brussels-Capital regions of Belgium, in areas with acceptable access to mental health care for refugees, participated in the study. Based on the information provided by school directors about their current student population, as well as the availability of at least two translators per language, only refugee minors aged 14 to 18 who were able to read and write in Pashto, Dari, Arabic, Dutch, French or English were included. This resulted in 141 participants. Questionnaires in which students choose not to answer the questions about NSSI were considered invalid (n = 15), as were those by students not complying with the aforementioned age restriction (14 to 18 years old) (n = 5).

Recruitment started in February 2017. From May to July data were collected within the school’s classrooms and during school hours, over a regular 50 min class period, under supervision of the first author and in the presence of at least one member of the school team, known to the students. All communication and questionnaires were translated and back-translated in Arabic, Dari, Pashto, English, French, and Dutch. Participation was voluntary. No questions were asked concerning grounds for refusal. Informed consents were obtained from all school directors and participants. All parents and guardians were informed about the study and the ability to end participation. Contact details of mental health services were provided to everyone involved. Following the data collection, referral to mental health services was requested for only one participant, known to the school for self-injurious behaviour, drug abuse and exhibiting psychotic symptoms. To date, no extra aftercare has been requested.

### Participants

The majority came from Syria (29.8%, n = 36) and Afghanistan (28.9%, n = 35). Participants from other countries came in smaller proportions. Due to their better representation, this study only compared Afghan and Syrian students to identify possible differences between NSSI and country of origin. The mean age was 16.12 years (SD = 1.23; range 14–18 years). More sample characteristics can be found in Table 1. Within the group of accompanied minors, 66% lived with both parents (n = 62), 18.1% lived with their mothers only (n = 17), 7.4% lived with their fathers in the absence of their mothers (n = 7), and 7.5% lived with family other than their parents (n = 6). On average, participants have been living in Belgium for 12.39 months (range 1–29 months, SD = 6.56).

**Table 1:** Sample demographic characteristics

	M	SD
Age	16.12	1.233

	n	%
*Country of origin*		
Syria	36	29.8
Afghanistan	35	28.9
Iraq	10	8.3
Somalia	11	9.1
Other	29	23.9
*Gender*		
Male	73	60.3
Female	48	39.7
*Family presence*		
Accompanied	95	78.5
Unaccompanied	26	21.5
*Legal status* (12 missing values)		
Asylum seeker	31	28.4
Recognized refugee	40	36.7
Family reunion	32	29.4
Other	6	5.5
*Housing situation* (8 missing values)		
Asylum center	17	15
LOI (translated: Local Housing Initiative)	4	3.5
House/apartment with 1 or more family members	75	66.4
House/apartment: alone	8	7.1
With a foster family	1	.9
With a friend	1	0
Other	7	6.2
*Religiosity* (2 missing values)		
Religious	113	95
Not religious	6	5
*Religion* (3 missing values)		
Christian	4	3.4
Muslim	105	89
Other	3	2.5
Not religious	6	5.1

### Measures

Participants were asked to complete a series of questionnaires. Closed-ended questions were used to measure socio-demographic data (e.g. age, gender, country of origin, date of arrival in Belgium, family structure, parental presence, housing situation, legal status, etc.).

The prevalence, methods, functions, and previous need for medical treatment, as well as the recency, frequency and future probability of self-injurious behaviour in community populations, were assessed via the ‘Screeningsvragenlijst opzettelijk zelfverwondend gedrag’, (translated: screening questionnaire intentional self-injurious behaviour) [42]. It uses 11 multiple-choice questions (e.g. ‘Have you ever hurt yourself on purpose in any of the following ways, without the primary intention to take your own life?’). This questionnaire was built around the DSM-5 symptoms for non-suicidal self-injury and is based on ‘The Brief Non-Suicidal Self-Injury Assessment Tool’ (BNSSI-AT) developed by Whitlock en Purington [43] for ‘The Cornell Research Program on Self-Injury and Recovery’. A question about the timing of the self-injuring behaviour (‘When was the first time you intentionally hurt yourself: before your flight, during your flight, or after arrival in Belgium?), as well as additional questions from the BNSSI-AT about functions, wound locations, circumstances, age of onset, initial motivations, and interference with daily life were added. For the Dutch version, the translation by Baetens and Claes [44] [‘De verkorte opzettelijk zelfverwondend gedrag vragenlijst’ (v-ZVGV)] was used. For all other languages, interpreters were hired for translation and back-translating, starting from the Dutch version. A study amongst an American community population of university students supports the reliability and the validity of the NSSI-AT, with alpha’s ranging from .38 to .66 [45]. No studies have been found to confirm these psychometric properties either for the BNSSI-AT or the ‘Screeningsvragenlijst opzettelijk zelfverwondend gedrag’, or for a population closer to the adolescent refugees as studied in this research.

The self-report version of the Strengths and Difficulties Questionnaire (SDQ) by Goodman [46], with impact supplement, was added to the battery. This was done to prevent non-self-injurious participants from distinguishing themselves too obviously from the self-injurious group by finishing too quickly. It also enabled us to obtain extra information about the emotional and behavioural difficulties experienced by the participants. As the SDQ, for children between three and 17 years old, is freely available online in several languages, including the languages used in this study (http://sdqinfo.org), the official translations were used. The 25 items of the SDQ can be divided into five scales, each consisting out of five questions. They screen for (1) emotional symptoms, (2) conduct problems, (3) hyperactivity and inattention, (4) peer relationship problems, and (5) pro-social behaviour, within the past 6 months. For example, the item ‘I have one good friend or more’ is one of the five questions screening for peer relationship problems. Each item is rated on a three-point Likert scale [47]. In the supplement, the adolescents are asked whether they believe they encounter difficulties in the areas of emotions, concentration, behaviour or being able to get on with other people, and if so, whether this implicates social impairment or a burden to others. Combined scores of the supplement generate an impact score of stress and impairment ranging from 0 to 10. Results were compared to three-band threshold scores, proposed by Goodman [46]. Goodman divided the normative population, based on a UK community sample, into a ‘normal’ group of 80%, a ‘borderline’ group of 10%, and an ‘abnormal’ group of 10%. For the normal group, clinically significant problems are unlikely; whereas a borderline score may reflect them. There is a substantial risk of clinically significant problems in the event of ‘abnormal’ scores [48]. High scores on the pro-social scale reflect strengths, all other scales measure weaknesses [47]. The validity and reliability of the self-report version of the SDQ ranges from satisfactory to good within a general European school population aged between 12 and 17. The psychometric qualities of the SDQ have also been confirmed for a Dutch community sample of children aged nine to 15, exhibiting an acceptable internal consistency (mean Cronbach’s alpha was .64) and test–retest stability, as well as good concurrent validity [49]. Even though the SDQ is available in many languages, one must bear in mind that its normative data are based on Western youths, complicating the interpretation for a non-Western refugee sample. Goodman and colleagues [50] examined SDQ data from seven countries and caution that “cross-national differences in SDQ indicators do not necessarily reflect comparable differences in disorder rates”. Exploring the reliability and validity of the SDQ and other measures, and introducing norms for a refugee population, if the heterogeneous nature of this ‘group’ would allow such a mission, could strongly improve the quality of research in this understudied field.

At the time of the study, 141 students of the 233 originally deemed eligible by the schools’ principle participated. Reasons for not completing the questions were (1) an inadequate level of reading or writing in the native language (n = 28), (2) not being present due to absence or activities outside of the school facilities (n = 50), (3) refusal to participate by the students (n = 12) or (4) by the parents (n = 2). Students were not obliged to fill in all questions. Of the 141 participating students, 121 questionnaires were considered valid for data analysis in SPSS (IBM SPSS Statistics Version 24). Non-parametric Chi square tests were used as well as independent-sample t-tests and Fisher’s exact tests for statistical comparisons of the obtained data.

### Ethical committee

This study is approved by the ethical committee of the university hospital of Brussels (Commissie Medische Ethiek UZ Brussel). However, given the assumed vulnerability of refugee minors, the committee added the following extra conditions. The minimum age of participation had to be 14 years instead of the originally intended 11 years of age. A member of the school team had to be present during completion of the questionnaires. Approximately a week after the questionnaires were completed, a second visit to the participating schools had to take place to ensure appropriate referral where necessary. Finally, an intermediate report had to be sent to the ethical committee after visiting the first participating school. All conditions were taken into account.

## Results

### Prevalence

Of all participants (*N *= 121), 17.4% reported a history of NSSI, with a 12-month prevalence of 11.4% (*n *= 17) (cf. Table 2). Out of the 21 participants who previously engaged in NSSI, seven came from Afghanistan (33.3%), six (28.6%) from Syria, and one from Iraq, Somalia, Albania, Iran, Congo, Burundi, Romania and Bangladesh (4.8% each). No significant differences between girls and boys were found concerning the lifetime prevalence (*χ*^*2*^ (1, *N* = 121) = .671, *p* = .413), nor for the average 12-months prevalence (*χ*^*2*^ (1, *N* = 114) = .147, *p* = .701), and the age of onset (*t*(17) = 1.42; *p* = .173; *d *= .65). There was no significant difference in the proportion of Afghan versus Syrian students concerning their engagement in NSSI (*χ*^2^ (1, *N *= 71) = .132, *p *= .717), their average 12-month prevalence (Fisher’s Exact Test (*N *= 64), *p *= .614), or their age of onset (*t*(13) = .733, *p* = .477, *d *= .41). Likewise, when comparing accompanied and unaccompanied minors, no significant differences in lifetime prevalence (*Fisher’s Exact Test* (*N* = 121), *p* = .154), 12-month prevalence (*Fisher’s Exact Test* (*N* = 114), *p* = .705) or age of onset (*t*(17) = .254; *p *= .803; *d *= .12) were found.

**Table 2:** Overview of lifetime prevalence, 12-month prevalence and age of onset

	All participants (N = 121)	Boys (N = 73)	Girls (N = 48)	Accompanied (N = 95)	Unaccompanied (N = 26)
Lifetime Prevalence	17.4%	15.1%	20.8%	14.7%	26.9%
12-Month Prevalence	11.4%	10.4%	12.8%	10.8%	14.3%
Age of onset (years)	13.11 (SD = 2.31)	13.80 (SD = 1.99)	12.33 (SD = 2.50)	13.00 (SD = 2.30)	13.29 (SD = 2.50)

When looking upon the number of times a person hurt him- or herself in the past, 68.4% reported more than five acts of NSSI. Living with or without both parents had no significant influence on NSSI (*χ*^*2*^ (1, *N* = 121) = 3.261, *p* = .071), nor had living in an asylum centre (*Fisher’s Exact Test* (*N* = 121), *p* = .734).

### NSSI methods

In terms of variability, refugee minors engaging in NSSI used an average of 2.65 methods (*SD *= 2.50, range 1–9). The majority applied only one method (55%), 20% five or more methods. Scratching was most commonly used (55%), followed by banging or punching objects (40%) and banging or punching oneself (30%), as shown in Table 3. The most commonly injured areas were hands (n = 13), wrists (n = 7) and arms (n = 7), regardless of gender.

**Table 3:** Methods of NSSI used according to gender

Method	All (N = 21) (%)	Boys (n = 11) (%)	Girls (n = 10) (%)
Scratching	55	45.5	66.7
Banging or punching objects	40	45.5	33.3
Banging or punching oneself	30	36.4	22.2
Cutting	25	18.2	33.3
Carving	25	27.3	22.2
Burning	20	18.2	22.2
Biting	15	9.1	22.2
Preventing wounds to heal	15	18.2	11.1
Pulling out hair	15	0	33.3
Other methods	20	9.1	33.3

### Functions

The mean number of functions per person was six (*SD *= 4.97, range 0–16) with no significant difference between boys and girls (*t*(18) = − .351; *p *= .729; *d *= .16), countries of origin (Afghanistan vs. Syria) (*t*(11) = − 2.086; *p *= .074; *d *= 1.19), or accompanied and unaccompanied minors (*t*(18) = − .184; *p *= .856; *d *= .09). Most reported were the automatic functions of practising suicide (*n *= 13, 72.2%), coping with uncomfortable feelings (e.g. depression, anxiety) (*n *= 12, 66.7%), and relieving stress or pressure (*n *= 9, 50%). This top three remains unchanged when looking at the boys separately. For the girls, the third most tagged function of NSSI is dealing with anger (*n *= 5, 50%) after coping with uncomfortable feelings (*n *= 6, 60%) and to the same extent as practising suicide (*n *= 5, 50%). For 55% of students with a history of NSSI, (practising) suicide was the primary intention, but never the sole function.

### Strengths and difficulties (SDQ)

Within our refugee sample ‘abnormal’ scores were observed for 16.2% of the participants on the Total Difficulties Scale, for 18.6% on the Emotional Problems Scale, for 10.6% on the Conduct Problems Scale, and for 2.7% on the Hyperactivity Scale. On the Peer Problems Scale, 15.7% of the participants scored ‘abnormal’. As little as 4.2% of the refugee minors had ‘abnormally low’ scores on the Pro-social Scale, measuring their strengths. Finally, 27.4% scored ‘abnormal’ on the Impact Scale, indicating the high self-perceived impact of their problems on their environment.

**Fig. 1:**
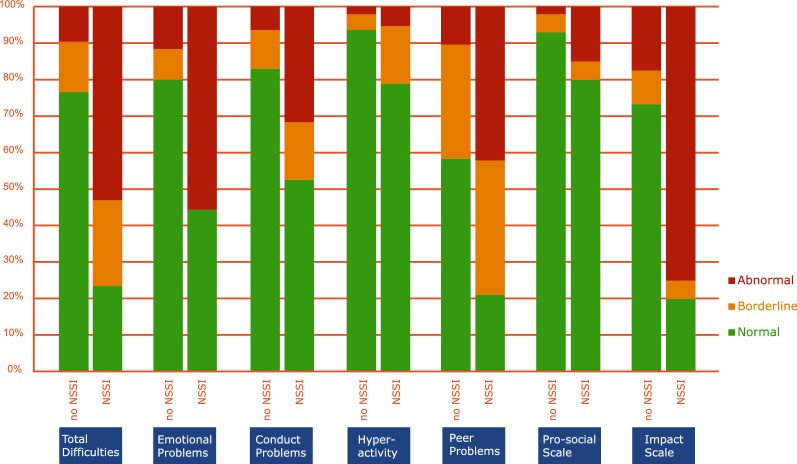
Three-band threshold scores applied on the SDQ results. The 3-band threshold scores, as proposed by Goodman [46] applied on the SDQ results of NSSI group and non-NSSI group for all scales. The original thresholds divided the normative population, based on a UK community sample, into a ‘normal’ group of 80%, a ‘borderline’ group of 10%, and an ‘abnormal’ group of 10%

NSSI participants differ significantly from their non-injuring counterparts on all scales of the SDQ but one, the Hyperactivity Scale, as shown in Table 4. When only those students with a history of NSSI were taken into consideration, there was a significant difference in conduct problem scores between those who engaged in NSSI during the past year (66.7% abnormal, 11.1% borderline) and those who didn’t (0% abnormal and 33.3% borderline); Fisher’s Exact Test (N = 15) = 6.627; p = .048. No other significant differences were found in scores between these two groups. Figure 1 offers a visual overview of the proportions in which the NSSI-group and the non-NSSI-group report ‘normal’, ‘borderline’ or ‘abnormal’ results in comparison to the Western normative population, as indicated by R. Goodman [46].

**Table 4:** Overview of the proportions in which groups report ‘abnormal’ results on the SDQ

	Abnormal score in general (%)^a^	Abnormal score for participants with a history of NSSI	Abnormal score for participants with no history of NSSI	Difference between NSSI and no NSSI groups
Total Difficulties Scale	16.2	52.9	9.6	*p *= .000
Emotional Problems Scale	18.6	55.6	11.6	*p *= .000
Conduct Problems Scale	10.6	31.6	6.4	*p *= .004
Hyperactivity Scale	2.7	5.3	2.1	*p *= .084
Peer Problems Scale	15.7	42.1	10.4	*p* = .001
Pro-Social Scale	4.2	15	2	*p *= .48
Impact Scale	27.4	75	17.5	*p* = .000

^a^Goodman [46] divided the normative population, based on a UK community sample, into a ‘normal’ group of 80%, a ‘borderline’ group of 10%, and an ‘abnormal’ group of 10%

Within the SDQ, boys and girls only significantly differed in emotional problems and conduct problems with 34.1% of girls reporting an ‘abnormal’ grade (‘borderline’ = 2.3%) of emotional problems versus 8.7% of boys (‘borderline’ = 10.1%) (Fisher’s Exact Test (N = 113) = 12.19, p = .002). We noticed a significantly larger proportion of conduct problems in males (‘borderline’ = 17.4%; ‘abnormal’ = 10.1%) compared to females (‘borderline’ = 2.3%; ‘abnormal’ = 11.4%); *χ*^*2*^ (2, *N *= 113) = 6.04, *p *= .049. Comparing origins (Afghan versus Syrian) for their strengths and difficulties only gives a significant difference on the Total Difficulties Scale with 26.5% of Syrians reporting ‘abnormal’ amounts of difficulties (‘borderline’ = 11.8%) as opposed to 5.9% of Afghans (‘borderline’ = 29.4%); *χ*^*2*^ (2, *N *= 68) = 7.05, *p *= .029.

On the impact scale, indicating the high self-perceived impact of their problems on their environment, unaccompanied refugee minors score significantly higher (52% score ‘abnormal’) than their accompanied peers (20.7% score ‘abnormal’): *χ*^*2*^ (2, *N *= 117) = 11.07, *p* = .004. On all other scales no significant differences between the two groups were found.

## Discussion

This study is an attempt to fill a void in the existing literature by exploring NSSI prevalence, methods, and functions in refugee minors in Belgium.

Contrary to expectations, refugee minors do not differ greatly from their Western counterparts in prevalence, methods or functions of NSSI behaviour. A lifetime prevalence of 17.4% was measured and is comparable to the 17.2% and 18% found by Swannell et al. [30], and Muehlenkamp et al. [29]. The 12-month prevalence of 11.4% is in accordance with the 9.6% to 28.4% found by Swannell et al. [30]. The resilience approach, allowing different mental outcomes when experiencing similar risks, may serve as a plausible explanation [25], combined with the hypotheses that people with certain mental disorders are less likely to succeed in their search for safer grounds [26]. However, 68.4% of self-injurers reported five or more acts of NSSI, indicating a greater psychological burden, as this number fluctuates around 25% in studies on Western adolescents [33, 34]. The age of onset of 13.11 years falls within the 12 to 14 years found for a Belgian and Dutch adolescent population [7, 35]. As in most literature on Western samples [e.g. 30], no statistical differences were found between boys and girls relating to lifetime or 12-month prevalence, or age of onset. Girls did report significantly more emotional problems and boys addressed more conduct problems (cf. SDQ). Being accompanied or not, having both parents around, or living in an asylum centre, did not influence the prevalence of NSSI. These findings are in accordance with research by Bean et al. [12], where very little variance in mental health outcome for refugees was found when examining gender, presence of family, and living in a centre. Unaccompanied refugees did, however, estimate the self-perceived impact of personal problems in their different life domains (i.e. home life, friendships, classroom learning, and leisure activities) to be higher in comparison to their accompanied peers (cf. SDQ Impact Scale).

Currently, a common idea among Belgian caretakers working with refugees is that Afghan males engage more in NSSI than other refugees do. Within this study however, no statistical differences in NSSI prevalence were found between Syrian and Afghan refugee minors. Moreover, Syrians reported significantly more difficulties than Afghan refugees (cf. Total Difficulties Scale, SDQ). The popular belief may be partially explained by the fact that there are more Afghan than Syrian refugees in Belgium [51]. A higher presence could result in more visibility to those working with refugees and can create the misconception of more mental health issues. This might also account for the idea that unaccompanied minors as well as youths residing in asylum centres are thought to be more sensitive to self-injury, since in both cases more caretakers are involved. Unaccompanied youths are more closely monitored than children who are part of a family and it needs no explanation that people living in asylum centres have less privacy than those inhabiting houses.

Literature has shown that religiosity is a protective factor for NSSI [8, 21]. The results of this study show no significant differences in NSSI behaviour between religious and non-religious individuals. However, the proportion of non-religious people in this sample is small (5%) and the manner used to investigate the nature of religiosity (i.e. through the questions ‘Are you religious?’, and ‘If yes: Christian, Muslim, Hindu, or other…’) seems too limited to jump to conclusions. Furthermore, stating that one is religious does not indicate how this religion is experienced or put into practice.

The number of methods used per person is also comparable with Western adolescents: 55% restricts themselves to one method (vs. 39.8–47.75% in a Western population), 20% applies five or more methods (vs. the 11.26–22.8% of Western adolescents using four or more methods) [34–36]. There seems to be less difference concerning the choice of method between boys and girls compared to a Western population with both genders preferring scratching, and banging or punching objects.

The mean number of six functions per refugee engaging in NSSI is comparable to the 4.3 functions per Western adolescent reported by Zetterqvist et al. [4].

Similar to their Western peers [e.g. 27, 38], refugees mainly report automatic functions. The most reported function was that of practising suicide. Joiner’s Theory of Acquired Capability for Suicide hypothesises that NSSI can lead to suicide when there is social isolation and the belief of being a burden to others [39]. The data of the SDQ questionnaire (cf. Table 4) clearly indicate the high proportion of peer problems (e.g. being solitary, not having many friends, not being liked or being bullied). In combination with the high impact participants engaging in NSSI estimate that their problems have on different areas of their daily life (i.e. home life, friendships, classroom learning, leisure activities) (cf. SDQ Impact Scale), these findings might suggest their considerable vulnerability for committing suicide. However, this study did not focus on suicide. Suicide and practising suicide were two functions from a long list of functions to be ticked if applicable. Further research is needed to establish how suicidal ideation and NSSI are connected in relation to refugee minors.

Research by Klonsky and Olino [28] indicates that approximately one out of five young adults engaging in NSSI exhibit high clinical symptomatology. Even though the SDQ is only a screening instrument, its results suggest that refugees engaging in NSSI suffer from more emotional and behavioural problems than their Western peers; with more than 50% reporting abnormal levels (i.e. a considerable risk of clinically significant problems) of total difficulties and emotional problems, as well as increased levels of conduct and peer problems.

In order to discern whether Western adolescents and refugee minors can be treated for NSSI in a similar way, it is necessary to take a closer look at this behaviour within the refugee population. Several circumstances in the past, present and future could have predicted higher NSSI outcomes. However, this study did not find notable differences, possibly suggesting different coping mechanisms upon which treatment could be focused. Furthermore, it might be possible that certain cultures have higher or lower levels of acceptance towards behaviours like NSSI and suicide. More research is needed to understand these findings and to learn how people around the world look upon NSSI behaviour.

### Limitations of the study

Due to practical restrictions, only those students who were able to read and write in one of the six proposed languages (Dari, Pashto, Arabic, Dutch, French, or English) were admitted. The selection of languages was based on the information eligible schools provided on the literacy of their refugee student population at the time of the study. Inclusion of less literate students would only have been possible using qualitative methods (e.g. interviews), but could have caused additional problems. A higher prevalence of NSSI in studies based on self-reporting questionnaires (19.7%) than when participants were interviewed (6.8%) [30] must be taken into consideration for future studies trying to include both literate an illiterate refugee minors. This variation in prevalence could possibly be explained by the difference in levels of anonymity between both methods. Anonymity has been shown to be important when sensitive issues are being addressed [30].

For this study we chose to work with students who could read and write, but the ability to do so does not mean one is used to doing so. Some students seemed to have difficulties with the concept of a questionnaire, and had difficulty with seemingly trivial issues like how to tick a box and when to add an answer in writing. Future studies examining a refugee population should take this into account and should strive for simple wording in their questions.

Before completion of the questionnaires, students were asked to sign an informed assent form, which was attached to the questions. Even though students were informed about the fact that no names would be included in the data processing, this potential identification might have induced a social desirability bias. The presence of a schoolteacher known to the participants may have had the same result. Future studies could accomplish more anonymity by limiting the class presence to people not known to the students and by splitting informed assents from questionnaires.

Due to the choice of working through OKAN-schools, minors refusing to go to school were never addressed, nor were students who had been in Belgium long enough to be enrolled in regular Belgian education. Together with the above mentioned language and literacy restrictions, the choice of schools based on their proximity to mental health services and the minimum age of 14 years old, resulted in a relatively small convenience sample of 121 valid participants. Different schools also imply different testing circumstances in terms of class temperature, privacy, timing, et cetera. Absenteeism of students (possibly due to mental difficulties), refusal to participate, as well as the lower level of education of youths who were unable to participate, should be taken into consideration when interpreting prevalence numbers attained through this study.

The current study is based solely on student reporting. This can be considered a bias and could be solved by expanding the research with questionnaires for teachers, parents or guardians. However, this would again decrease anonymity. Moreover, the accuracy of adults assessing mental health in refugee minors is found to be unreliable, possibly due to differences in interpretations of the questions, the parents or guardians not being aware of the problems, and the judgment of when to label something ‘a problem’ [12].

Finally, for this study we chose to compare the results to the existing literature. It would be interesting for future research to involve a Western sample by means of control group and statistically compare both groups. With bigger sample sizes, it would also be interesting to statistically analyse the studied groups more detailed: how do unaccompanied girls compare to accompanied girls, etc.

### Implications of the study

This study stresses that refugee minors often feel socially isolated and a burden to others, indicating an increased risk for suicidal ideation [39]. Research by Mels et al. [52] suggests the importance of social support in controlling migration stress in unaccompanied asylum-seeking children. It might be interesting to investigate the link between social support and NSSI since social support could play a crucial role in the refugees’ wellbeing and possibly in their self-injurious behaviour.

As refugees do not always find the way to mental health services, and mental health services are not always accustomed to working with refugees, prevention seems to be the best way to addressing this problem. Enhancing social networks, but also enhancing the perceived meaning in life, and positive relationships with important others, preferably the parents, could reduce suicide risk and should be embedded in prevention programs.

For those acquiring therapy, Dialectical Behavioural Therapy for Adolescents (DBT-A) has been tested on a Western sample [53]. It reduces suicidality and NSSI behaviour. When trauma is involved, Eye Movement Desensitization and Reprocessing (EMDR) has also shown to be effective [54, 55]. More research is needed to explore the applicability of these treatments to refugee adolescents.

## Conclusion

The findings above hardly show any difference in NSSI prevalence, methods and functions between refugee and Western adolescents. This possibly suggests strong protective factors or different coping styles or self-regulation techniques in refugee minors. The findings do, however, indicate more repetitive forms of NSSI, a substantial risk of clinically significant problems in self-injuring refugees (i.e. high rates of abnormal scores on SDQ), and an increased risk of suicidal behaviour. More research is needed to explore protective factors, as well as the different reactions of refugees to stress and adverse life events. Research comparing refugees in less safe camps en route to Europe might also shed a light on the effects of arriving in a safe country of refuge.

## Abbreviations

BNSSI-AT: Brief Non-Suicidal Self-Injury Assessment Tool
DBT-A: Dialectical Behavioural Therapy for Adolescents
D-SIB: deliberate self-injurious behaviour
EMDR: Eye Movement Desensitization and Reprocessing
LOI: Lokaal Opvang Initiatief (i.e. Local Housing Initiatives)
NSSI: non-suicidal self-injury
OKAN: Onthaalklassen Anderstalige Nieuwkomers (i.e. reception classes non-Dutch speaking newcomers)
SES: socioeconomic status
SDQ: Strengths and Difficulties Questionnaire
v-ZVGV: verkorte opzettelijk zelfverwondend gedrag vragenlijst
